# Factors affecting aerial spray drift in the Brazilian Cerrado

**DOI:** 10.1371/journal.pone.0212289

**Published:** 2019-02-19

**Authors:** Fabio Henrique Rojo Baio, Ulisses Rocha Antuniassi, Bruno Rodeguer Castilho, Paulo Eduardo Teodoro, Eder Eujácio da Silva

**Affiliations:** 1 UFMS - Chapadão do Sul Campus, Chapadão do Sul/MS, Brazil; 2 Unesp/FCA - Botucatu, São Paulo, Brazil; Julius Kuhn-Institut, GERMANY

## Abstract

Pesticides aerial application may results in the drift to neighboring areas if some application technology is not well executed. This phenomenon should be minimized to reduce environmental risks and agricultural production costs. This work aimed to investigate the interaction of environmental conditions, surrounding distance, and application conditions influencing spray drift in aerial applications. Sampling data from aerial sprays were collected during three agricultural years (from 2012 to 2014) in fields cultivated with sorghum, millet, soybean, corn, and cotton. The following variables were evaluated: application swath width, application rate, distance from the applied field, wind speed, relative humidity, and temperature. The estimated Pearson’s correlations and path analysis identified that application rate and distance from the applied field and application were the variables that most influenced drift. Equations relating spray drift in function of distance from the applied field and application rate were adjusted in function of the variable, and a response surface model was constructed to estimate drift. The major pesticide class sprayed with aircraft in the Brazilian Cerrado was insecticide, followed by fungicide. This scenario shows the potential hazard risk of spray drift over the environment. The concentration of the drift deposits decreased as surrounding distance and application rate were increased. A mathematical equation of drift prediction was established, where the variables that contributed most to drift deposits were surrounding distance and wind speed. Thus, it is very important to monitor and respect the wind speed limits during the aerial spraying, mainly when there is any risk potential associated with pesticide exposure over the downwind direction.

## Introduction

Pesticide application corresponds to a most considerable portion of the production cost with annual crops. The concern about the use of these products in agriculture has significantly increased in recent years, especially regarding human health and environmental contamination [1]. This issue is persistent not only in rural but also in urban areas. One of the leading causes of contamination is linked to the transport of pollutants from agricultural areas by air, water, or another natural resource.

Application technology aims to efficiently and economically apply the correct amount of the active ingredient to the desired target, without affecting the environment [2]. Several parameters must be considered when applying a product, such as a target, the spray application rate (SAR), the product physicochemical properties, environmental fate, and toxicology profile. Aerial application is a valuable tool when used with clear technical criteria. Drift is one of the main reasons for product losses and consequent environmental contamination [3]. Spray drift is the amount of product that did not reach the target during spray application [4].

Aerial spray applications can cause drift to neighboring areas if the operation is not well performed. Since the application rate used in this method is reduced, usually lower than 10 L ha^-1^, proper target coverage is more difficult [3]. Therefore, additional strategies are required to ensure proper spray deposition while mitigating spray drift to the surrounding environment. In addition, droplets are more concentrated in aerial applications, which could increase the risk associated with pesticide drift depending on the active ingredient and the target [5].

Some authors have modeled spray drift as influenced by downwind distances from the application area. However, relatively shorter distances were evaluated [6,7]. There is a direct interaction between drift and wind speed [8]. The wind speed must be measured in any drift evaluation experiment, mainly by the direct use of an anemometer at the application time. Also, it is important to characterize the location where the drift measurement will be performed. At least one weather station shall be provided for measurement of meteorological conditions, located within 30 m of the spray swath [9], acquiring temperature, wind direction and relative humidity of the air. During spray application, a considerable amount of the total pesticides applied can be lost to the air in the form of drift [6].

Determining spray drift deposition at different downwind distances is complex as many factors are involved in the process. Several factors can influence spray drift in aerial applications, such as wind speed, spray solution, flight speed, flight height, boom setup, nozzle selection, spray pressure, deflection angle, and distance from the boom sprayer to the target [10].

To facilitate the measurement process of drift deposits, some researchers have collected these deposits on a drift test bench [5,8] or wind tunnels [11]. Despite their advantages, these indirect methods cannot reproduce all real aerial application conditions and complete drift studies must be conducted in the field [12].

Aerial spray drift can be measured on the ground at different distances from the applied field, using passive or active sampling collectors [12], but a large amount of personnel and time resources is required. Active drift collectors force the air flux to pass by a filter, which is analyzed in the laboratory. Passive collectors are the most commonly used equipment for drift measurement techniques, both for ground and airborne spray drift. In these studies, a tracer dye is frequently used as a chemical reference for measurement of the spraying syrup deposition. In order to decrease de complexity of working with tracer dye in the spray drift field experiments, Lidar sensors can be used to directly measure the spray drift pattern [13], but the cost of this technique is higher.

This work aimed to investigate the interaction of environmental conditions, surrounding distance, and application conditions influencing spray drift in aerial applications.

## Material and methods

### Experimental area

Sampling data from aerial sprays were collected in sorghum (*Sorghum bicolor*), millet (*Pennisetum glaucum*), soybean (*Glycine max*), corn (*Zea mays*), and cotton (*Gossypium hirsutum*) fields during three agricultural seasons (2012–2014) in northern Mato Grosso do Sul, Brazil (approx. lat. 18.0° - 19.1° S; long. 52.4° - 53.6° W). ASABE standard was used to standardize the experiment [9]. The experimental fields are in the Cerrado biome. The climate is Aw [14], or tropical climate with dry winter. The topography is predominantly flat to gently rolling, and 78% of the area presents less than 5% slope. The research was conducted in experimental area under the responsibility of Dr. Fabio H. R. Baio. The research was conducted with the permission of the farmers of this experimental area. In this study there are no endangered or protected species or locations.

### Characterization of aerial sprays and aircraft

Twenty-five pesticide aerial applications were carried out and had their drift potential evaluated. The fields totaled an area of 80 and 200 ha, requiring at least two aircraft refilling for the total application in each field. The aircraft tank was filled with a single pesticide or a mix of pesticides, used to control the respective pests and diseases of the crops of each field. Of the twenty-five aerial applications, tank syrup from five of them contained only the tracer dye. The spray application rate varied from 10 to 40 L ha^-1^ and the application swath width varied from 15 to 23 m.

The applications were performed by an EMB-202 Ipanema model agricultural aircraft, from Embraer [15], with 7.43 m in length, 2.22 m of height, 11.47 m of wingspan, and 194 kW (260 hp) engine. Applications were performed at 160.9 km h^-1^, with a flight height between 4 and 5 m above crop canopy, according to the swath width. The aircraft spray boom was equipped with eight rotary atomizers Micronair AU5000 model [16], with propellers angle adjustment at 350 position, configured to generate 150 μm Volume Median Diameter (DMV) droplets. In all applications, the total spray volume in each aircraft load was 600 L.

Each application had a different operational condition, and the spray applications rates (10 to 40 L ha^-1^) varied according to the technical recommendation for each field. Application swath widths (ASW) varied from 15 to 22 m, based on each field, and were guided by a light bar guide DGPS SatLoc, LiteStar model [17]. The environmental meteorological conditions were monitored by measuring the temperature, relative humidity, and wind speed, with a portable meteorological station Instrutemp, model ITWH-1080. Environmental conditions measurements were obtained every five minutes for thirty minutes before, during, and after each aerial application.

### Tracer dye added to the spray

Rhodamine B (C28H31ClN2O3, or also known as tetra-ethyl-rhodamine), a fluorescent dye distributed by Pcilbase [18] was used as a tracer for spray drift measurements at the equivalence of 800 mg ha^-1^ (the higher the SAR, the lower is the dye concentration, proportionally). Calibration curves were developed for each one of the 25 tank solutions. The tracer dye was added to the spray tank in a premix preparation, which was pumped to the aircraft hopper. To this end, a concentrated rhodamine B solution, stored in an amber flask container with 5 L of distilled water, was prepared in each application and kept under refrigeration until its use, avoiding the degradation of the tracer dye. Rhodamine B tracer dye can be efficiently used in evaluations of agricultural pesticides deposition of aerial spray drift [19].

A 20-mL sample of each final spray was taken from the aircraft hopper after the filling, allowing the preparation of the calibration curves used in the fluorometer, where the concentrations of drifted rhodamine were determined. This procedure used a Trilogy fluorometer, from Turner Designs [20], with a minimum detection of 0.02 ppb (parts per billion) of rhodamine.

### Field sampling points

Drift sampling points at different distances outside the applied field were used to collect the drift deposits. The positioning of the drift sampling points in each application was planned using the GIS software (Geographic Information System) SSToolbox 3.8. GIS was also used to generate drift maps, associating the concentration of the deposited tracer dye with the respective sampling point. The squared inverse distance methodology interpolated the values.

The geographic coordinates of the sampling points with the pre-defined distances from the center of the previously mapped field were exported to four GNSS collectors (2 units of Nomad, 1 Juno, 2 Geoexplorer—Trimble Navigation Ltd.) [21], allowing navigation to these points in the field. In the majority of the aerial applications, there were no terrain features or obstruction to airflow of the spray drift. On each sampling point, two passive drift collectors were placed outside the field and at six predefined distances from the border (10, 50, 200, 500, 1,200 and 2,000 m), in the four Cartesian directions, totaling 60 collectors in each application. However, in some cases, the predefined distance could not be used due to some physical impediment, such as a lake. When this problem occurred, the sampling point was placed in another site, maintaining the Cartesian direction, with a new georeferencing of the point. In four of the twenty-five aerial applications, the sampling points were positioned at a distance longer than 2,000 (maximum distance of 2,984 m) due to the existence of a physical impediment in the planned distance (sharp slope, or the presence of forest in the downwind path).

The sampling points positioned inside the field collected the aerial spray depositions on the crops’ canopy, enabling the establishment of a correlation with the tracer dye deposited in the external collectors. The positioning of these sampling points was planned using the GIS software and another GNSS Geoexplorer XM unit, previously characterized. Thirty sampling points were placed in a 15 x 100 m rectangular mesh in a central swath of the field.

### Characterization of passive drift collectors

**Fig 1:**
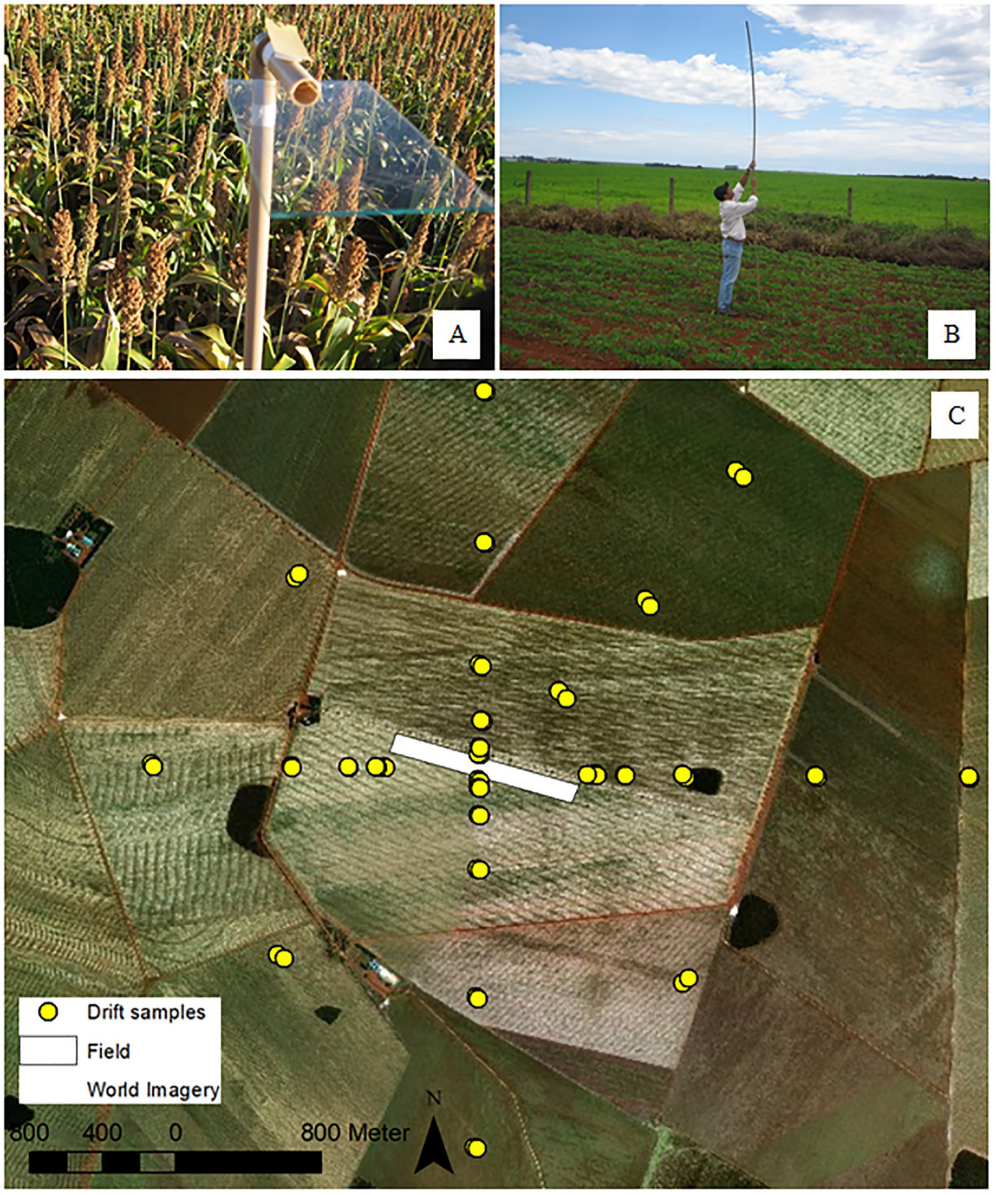
Positioning of the internal sample collectors for spray deposition collection (A), positioning of the external samples for drift collection (B), and samples point positioning (C).

The following passive drift collectors were used: polyamide (nylon) yarns with 1 mm diameter, positioned vertically at the external sampling points, and rectangular glass plates (0.017 m^2^), positioned horizontally at the internal sampling points (Fig 1A). A PVC plastic film was used to insulate the underside of the glass plates from the internal collectors, hindering droplets deposition on the underside. Two 2-m external collectors were positioned at 1.8 m from the ground and spaced at 10 m apart (Fig 1B). They were installed perpendicularly to the Cartesian line for each georeferenced sampling point (Fig 1C). The values of drift deposition used in the mathematical equation were obtained by the mean of the values measured in these two collectors. The internal collectors were placed immediately above the canopy.

### Sampling procedure

Samples collection (in-swath and surrounding area samples) only started at thirty minutes after each spray application, allowing the collection of the possible droplet spray in suspension on the air. The removal of the external collectors started from the shorter to the longer distances. Internal collectors were removed using disposable gloves, exchanged at each sampling point, packed in plastic containers with a lid, and stored in a cardboard box. External collectors were removed using disposable gloves and scissors (washed with ethyl alcohol) to cut the polyamide wires, which were packed in PVC tubes with a lid. This procedure aimed to avoid the contamination of the samples by the tracer dye.

Both the supports for the external and internal collectors were reused in the spray applications. Due to the low tracer dye concentration during the spray application, any remnants of this product from previous applications could compromise the drift quantification of subsequent applications. Thus, the supports were chemically decontaminated after each spray application by washing in running water with a detergent solution (Tween 80 at 1%) and using a cloth moistened with ethyl alcohol.

### Quantification of drift deposition

In the laboratory, both the external and internal collectors were washed with 30 mL solution of distilled water and nonionic surfactant at 1% v/v, Tween 80 from Sigma-Aldrich [22], allowing the extraction of the tracer dye from the collectors. Artificial targets are more efficient in the recovery of the tracer dye rhodamine B when compared with plant leaves [23]. The mass balance generated by the deposits of the tracer dye in the samples in relation to the initial concentration was used to calculate the deposition percentage from the aerial spray drift [19], by the following equation:

$$C_{i}V_{i}=C_{f}V_{f}$$ (1)

where: Ci = concentration of tracer dye in the mixture (μg L^-1^); Cf = concentration of tracer dye detected by the fluorometer (μg L^-1^); Vi = volume captured by target (mL); Vf = sample dilution volume (mL).

The concentration of the tracer dye Rhodamine B was estimated by fluorometric analysis using a Trilogy Laboratory Fluorimeter [20] and calibration curves. The internal collectors and the laboratory glassware were decontaminated by immersion in a saturated solution (30 g L^-1^) of potassium permanganate for five minutes, rinsed in running water, immersed in sulfuric acid and hydrogen peroxide solution (1:1) for five minutes, and then rinsed in distilled water.

### Statistical analyses

The data used for statistical analysis are contained in S1 Table. Boxplot was used to demonstrate the variation of the atmospheric and operational conditions graphically. The Pearson’s correlations were estimated between the evaluated variables, and the correlation network was used to express the results graphically. In this network, the proximity between nodes (traces) is proportional to the absolute value of the correlation between these nodes, where positive correlations were highlighted in green, and negative correlations were represented in red. Subsequently, a multicollinearity diagnosis was performed [24]. Path analysis was carried out considering drift as the primary dependent variable and the others as explanatory variables. However, to obtain the direct and indirect effects of path analysis, the correlation matrix must be well-conditioned. In the presence of multicollinearity, the variances associated with the path coefficient estimators can reach exceedingly high values, becoming unreliable. Also, parameters estimates may assume values outside the parametric space of r (> 1.0). Afterward, regression and response surface equations were adjusted using the variables selected by the path analysis. Finally, a decision tree was constructed as an alternative way to predict drift in function of the variables selected by the path analysis. In this process, 80% of the data were separated into a training set of the algorithm, and 20% was separated for the validation of the analyses. All analyses were performed in the free software Rbio [25].

## Results and discussion

**Fig 2:**
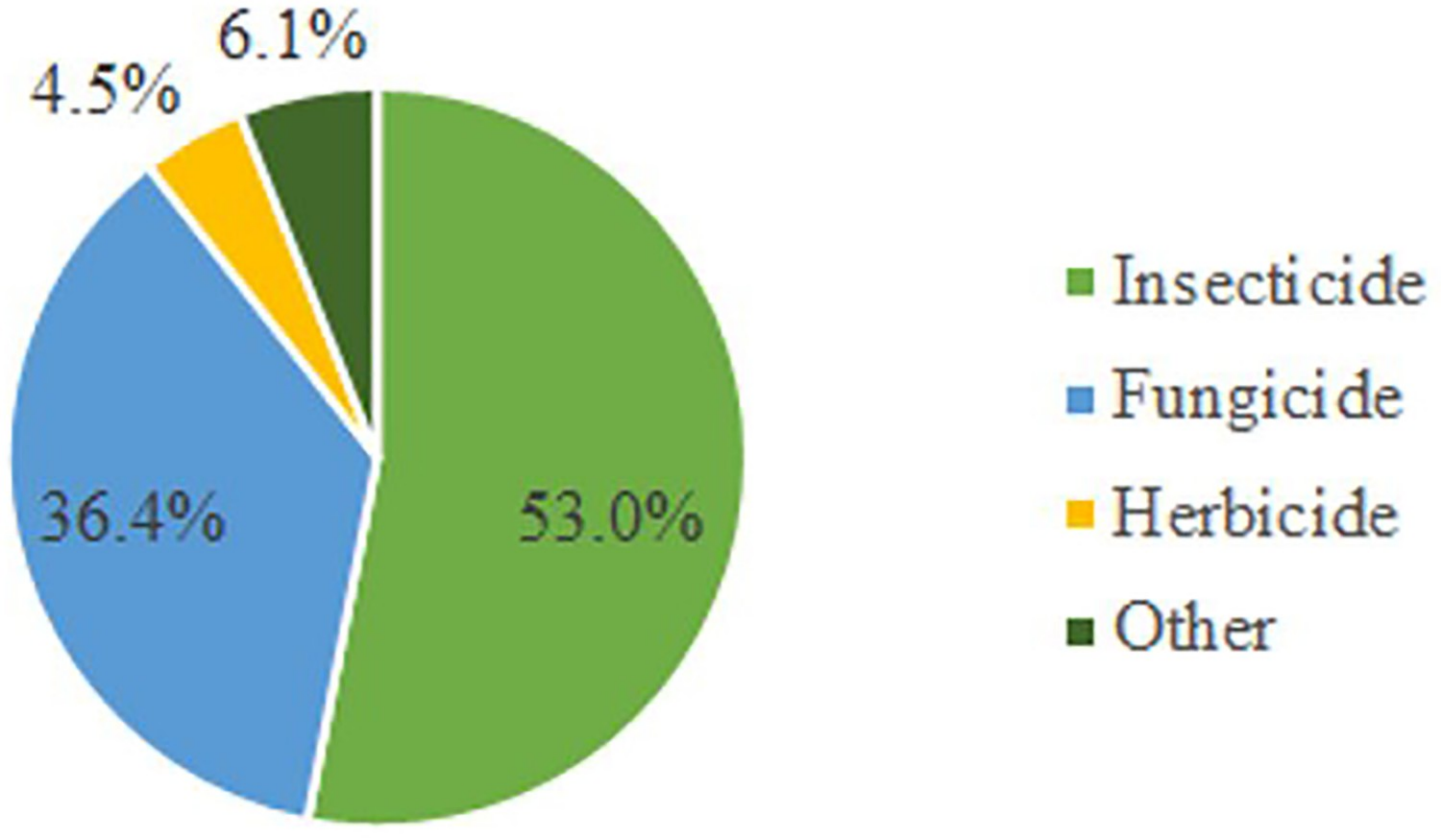
Classes of the pesticides applied over the sprayed areas.

Fig 2 illustrates the classes of the pesticides applied over the sprayed areas. The major pesticide class sprayed with aircraft was insecticide (53.0%), usually, the most hazard pesticide class, followed by fungicide (36.4%). The five most applied insecticides were acephate, diflubenzuron, thiodicarb, beta-cyfluthrin+imidacloprid, carbosulfan; presented at 51.5% of the syrup content. The five most applied fungicides were tetraconazol, triadimenol, metiram+piraclostrobina, epoxiconazol+piraclostrobine, carbendazim; presented at 70.8% of the syrup content. Only 4.5% of the syrup mixtures contained herbicide. Aerial applications of herbicide may cause issues especially when it is not properly performed [26]. Soybean was the crop with the higher aerial application use on this study (53.9%), followed by sorghum (19.2%), corn (15.3%), and cotton (11.5%).

**Fig 3:**
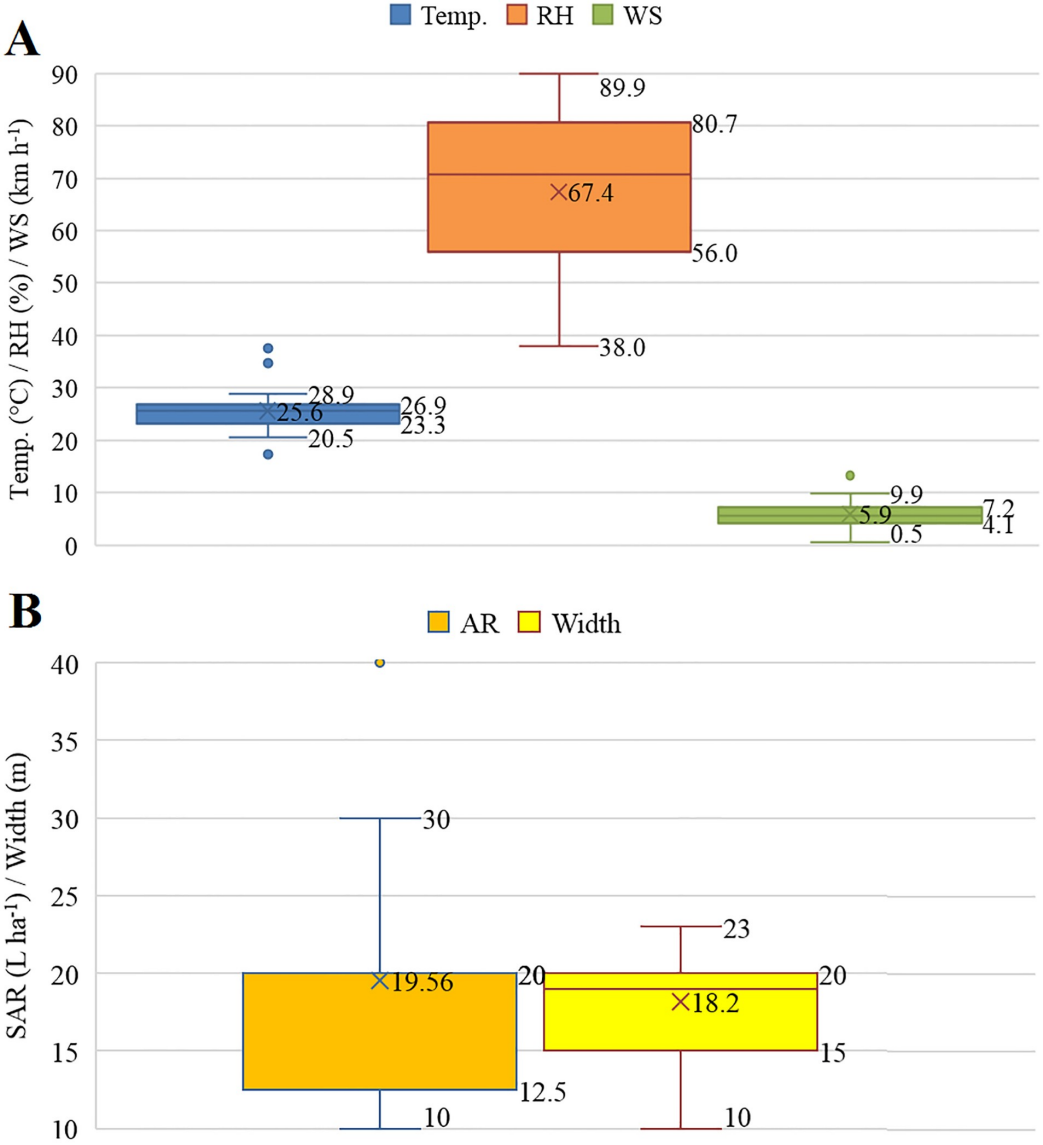
Variation of atmospheric (A) and operational (B) conditions during agricultural drift measurements (Temp.—Temperature; RH—Relative humidity; WS—Wind speed; SAR—Spray application rate; and width—Application swath width).

The measurements of the environmental conditions revealed that the most variable factor during the experimental period was the relative humidity (Fig 3A). The temperature had little variation during spray applications, with only three outliers. Wind speed exceeded 10 km h^-1^ in few application timings. The wind speed is the main environmental factor that affects aerial spray drift [6]. A significant variation was detected for other operational factors during spray applications, such as application rate and application swath width (ASW) (Fig 3B).

Environmental conditions are fundamental to the success of the aerial application technology, and in most cases, applications with relative humidity below 55% and ambient temperature higher than 30 °C should be avoided [27]. Ideally, applications should be performed with wind speed between 3 and 7 km h^-1^. The absence of wind can also be detrimental due to the occurrence of ascending heated air, hindering small droplets deposition. Several researchers consider that droplets smaller than 100 μm are easily carried by the wind, resulting in a greater drift potential [8].

A mass balance relation [19] was established between the concentrations of the tracer dye for the swath and surrounding spray deposition collectors of each field and for the different distances, using the same methodology of the tracer dye recovering for both collectors. Table 1 shows the mean values of tracer dye rhodamine B deposited on the internal collectors. The calculation of the mass balance of the application indicated a mean recovery of the tracer dye on the internal collectors of 48.6%, resulting in a mean drift of 51.4% of the total spray volume in all the monitored aerial spray application. The quantity of the spray deposited on crops can be significantly less than that released from sprayer [28]. Thus, further studies need to be applied to understand this scenario with a high amount of aerial spray drift and develop new application technology in order to decrease this rate. Also, the collection efficiency of the collectors can affect the tracer dye recovering result, and more studies on this scene need to be accomplished [29].

**Table 1:** Means of tracer dye rhodamine B depositions on the internal collectors.

Mean concentration of tracer dye Rhodamine B (μg m^-2^)	Internal mean deposition (%)
33.12	48.60

The mean of the total drift deposition on the external collectors (Table 2) decreased as surrounding distance increased. In general, the concentration of the tracer dye in the external collectors positioned at 10 m from the field border was 8.1%, and this proportion decreased as the distance increase up to 2000 m from the border of the applied fields. This deposition decrease along the different distances may be due to the droplet’s deposition along the downwind or evaporation. Other research relates deposition of the spray drift of aerial application at this distance from the field border in the order of 1.7% [6] to 18.0% [30].

**Table 2:** Means and percentages of depositions on external collectors in function of the different distances of all applications.

Distance (m)	Mean deposition ± standard errors (μg m^-2^)	Comparative deposition to internal collectors (%)
**10**	5.52 ± 2.97	8.1%
**50**	3.02 ± 0.84	4.7%
**200**	2.19 ± 0.41	3.3%
**500**	1.36 ± 0.25	2.0%
**1200**	1.09 ± 0.23	1.6%
**2000**	0.65 ± 0.20	0.9%

Drift depositions at the shortest distance from the field border account for most of the total drift. Some research likewise measured aerial spray drift deposition at distances of 320 m [31] up to 2,000 m [6] from the field border, measuring 0.10% and 0.05% of the dose applied, respectively. Analyzing the amount of spray drift found at 2000 m from the applied field, it can be discussed that a small portion of the spray syrup can be deposited even in a higher distance.

Previous studies reported that smaller droplets have lower weight and therefore are more subject to wind action since the droplet size directly influences the terminal speed [32] [33]. This fact hindered spray deposition, mainly when the aerial spray application uses droplets smaller than 200 μm.

**Fig 4:**
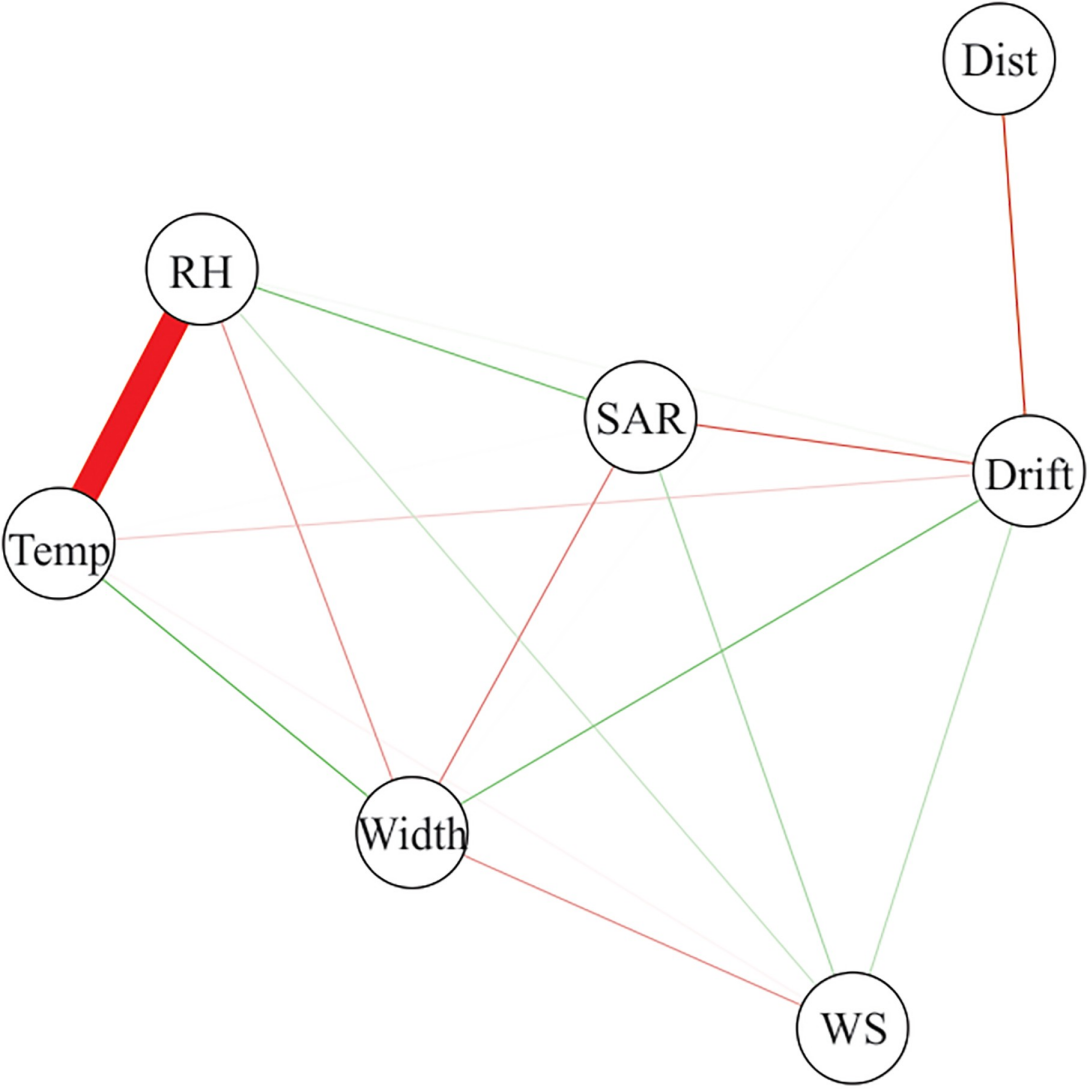
Correlation network between the measured factors and drift deposits (Dist: Distance; AR: Application rate; WS: Wind speed; RH: Relative humidity; Temp: Temperature).

The correlation network generated from Pearson’s correlation matrix revealed a strong negative relation between temperature and relative humidity when studied apart (Fig 4). However, these two variables little influenced the variation of drift deposits. Drift was moderately and negatively correlated with the distance from the field border and application rate. The wind speed and application swath width presented a moderate and positive correlation with drift. Some studies already proved that aerial spray drift is strongly affected by wind speed [6]. It was found that increasing the SAR decreased the spray drift. This scenario indicates that oil based adjuvants are commonly used in aerial application scenarios, and that when the SAR is reduced, generally the oil concentrations are increased, where drift could be potentialized [12, 13].

Despite its relevance, the Pearson’s correlation coefficient (r) may lead to misinterpretations about the relationship between two variables, and may not be a real measure of cause-and-effect. A high or low estimate of r between two variables may be the result of the effect that a third variable or a group of variables have on that pair, leading to the non-exact relative importance of the direct and indirect effects of these factors. Therefore, path analysis was carried out, which investigates the cause-and-effect relationship. This analysis provides detailed knowledge of the influences of each variable on the main dependent variable, discarding the effects of the others [34].

The matrix of correlation estimates presented severe multicollinearity, being necessary to add a constant k = 0.10 to the diagonal of this matrix to correct this effect [24]. Afterward, the condition number, given by the ratio between the highest and the lowest eigenvalue of the correlation matrix, was equal to 90, revealing weak multicollinearity.

**Fig 5:**
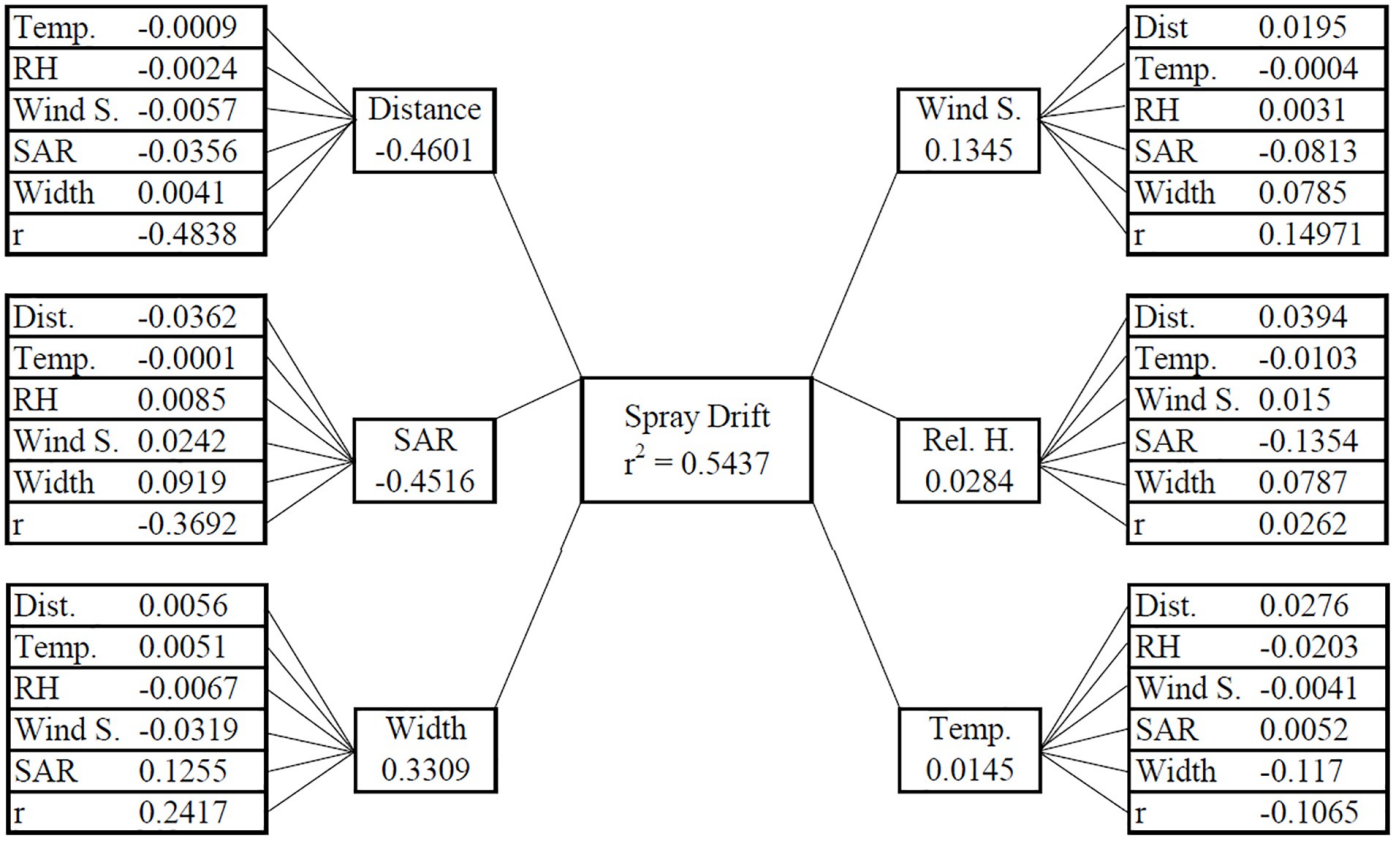
Path analysis with correlations of direct and indirect effects of the variables distance, application rate, application swath width, wind speed, relative humidity, and temperature of the air on spray drift.

The results of path analysis considering spray drift as the main dependent variable and the others as explanatory variables are expressed in Fig 5. The direct effects of each variable are represented in the primary boxes that derive from the main box, while the indirect effects of the other variables are represented in the secondary boxes. For a variable to have a cause-and-effect relationship with drift, it must present a high direct effect in the same direction as its Pearson’s correlation with drift. In this sense, the distance from the applied field and application rate are the main variables that influenced drift since the estimates of their direct effects were distance and SAR (direct effects: -0.4601 and -0.4516, respectively), and in the same direction and magnitude of their correlations with drift (r = -0.4838 and -0.3692, respectively).

According to the results, air temperature did not affect aerial spray drift. Results from other trials showed that droplet size and wind speed are the factors that most influenced drift distance [32]. There is a relationship demonstrating that by reducing the size of the drop, the rate of deposition decreases, increasing the time the drop takes to deposit on the target. Therefore, these droplets are more prone to evaporation and trajectory change, becoming more susceptible to the action of wind.

**Fig 6:**
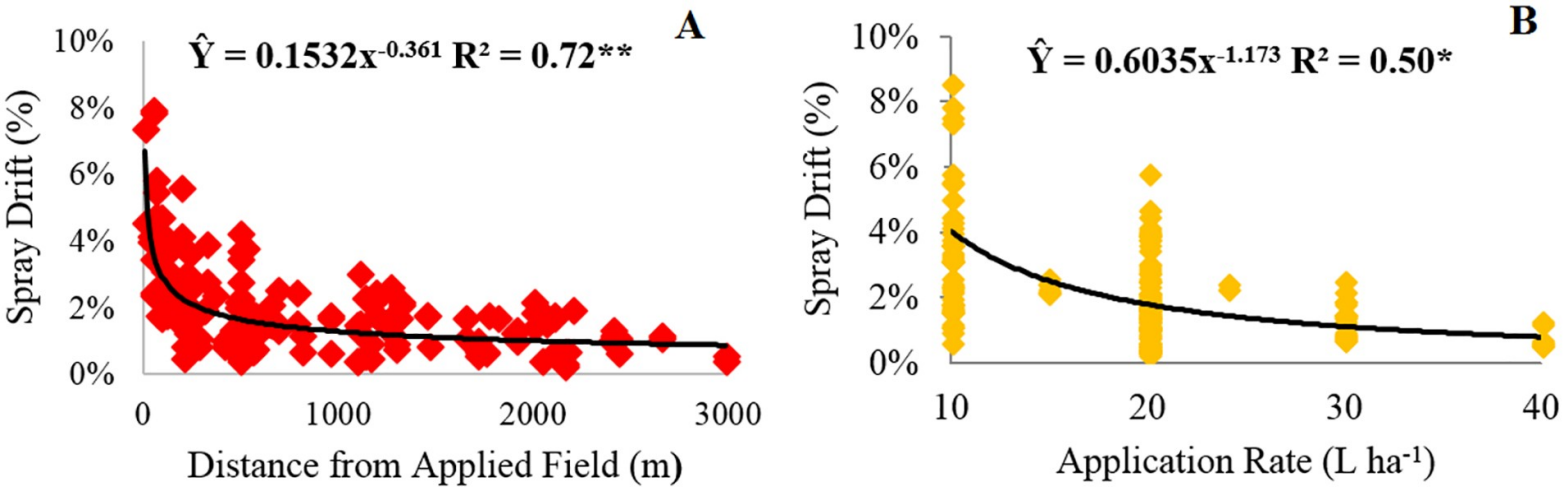
Spray drift (%) as influenced by distance from the applied field (m) and application rate (L ha^-1^). * and **: significant at 5 and 1% probability by the F test, respectively.

Surrounding distance and application rate were the most adequate variables to indirectly predict spray drift in this study conditions, as shown by path analysis. Thus, the adjustment of regression models considered these variables as explanatory (Fig 6). Both variables presented regression equation of the power type with the main dependent variable; however, the distance from the applied field (Fig 6A) was the variable that had a higher capacity to predict spray drift (R^2^ = 0.72).

**Fig 7:**
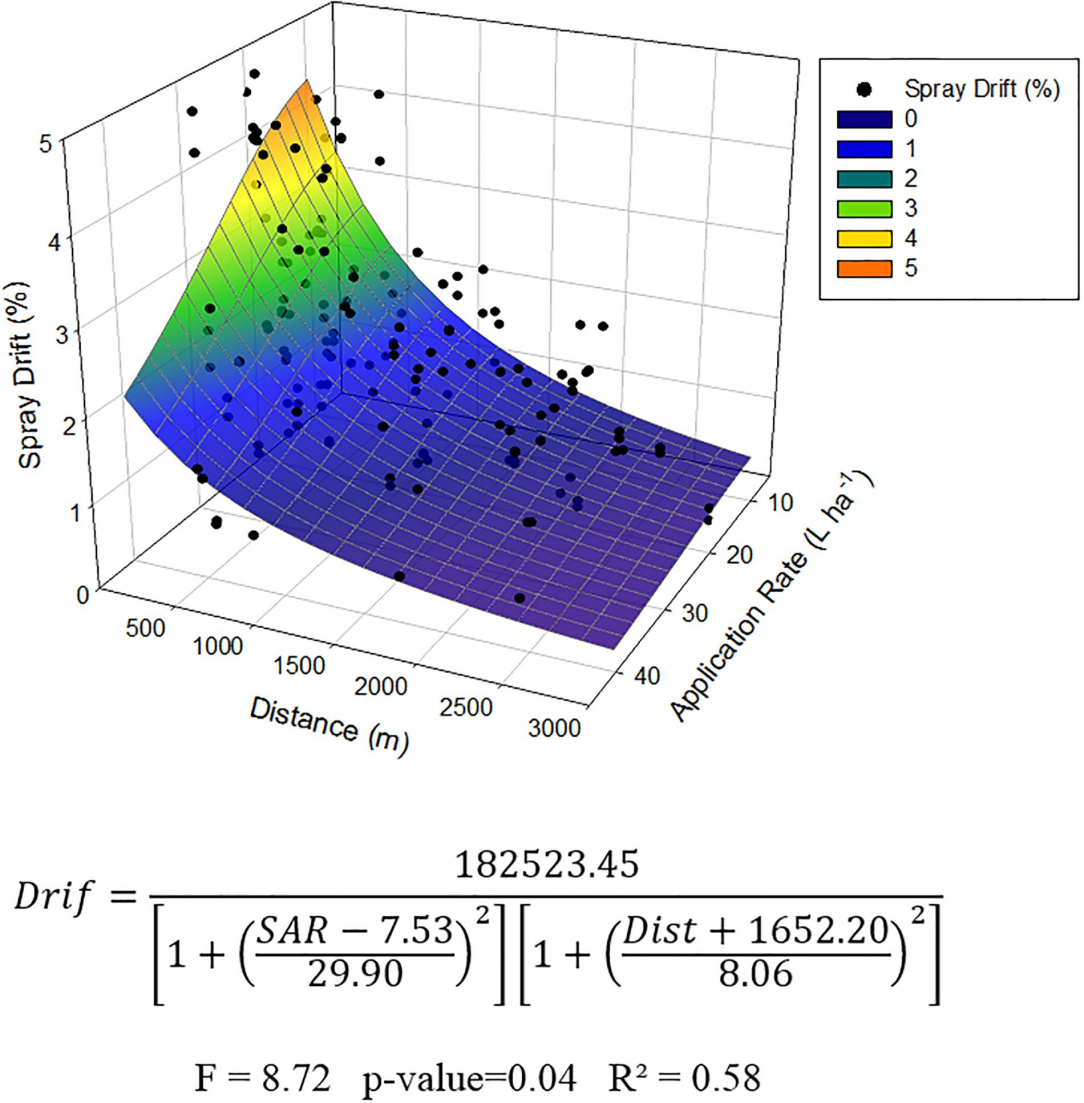
Response surface for drift deposits estimates in relation to application distance (Dist) and spray application rate (SAR).

The drift process in a spray application depends on several factors acting together. Thus, a response surface model was constructed considering simultaneously the distance from the applied field and the application rate to predict aerial spray drift, as shown in Fig 7. The adjusted equation was statistically significant and represented a quadratic surface. The concentration of the drift deposits decreased as the application rate was increased. In fact, when lower application rate is sprayed, a more concentrated syrup solution is used, then, probably, there is more concentration of the oil-based adjuvants that could increase drift [6]. Thus, every time that a lower SAR is applied, the aerial application became more technique, and more attention to the environmental condition need to be placed to minimize spray drift.

**Fig 8:**
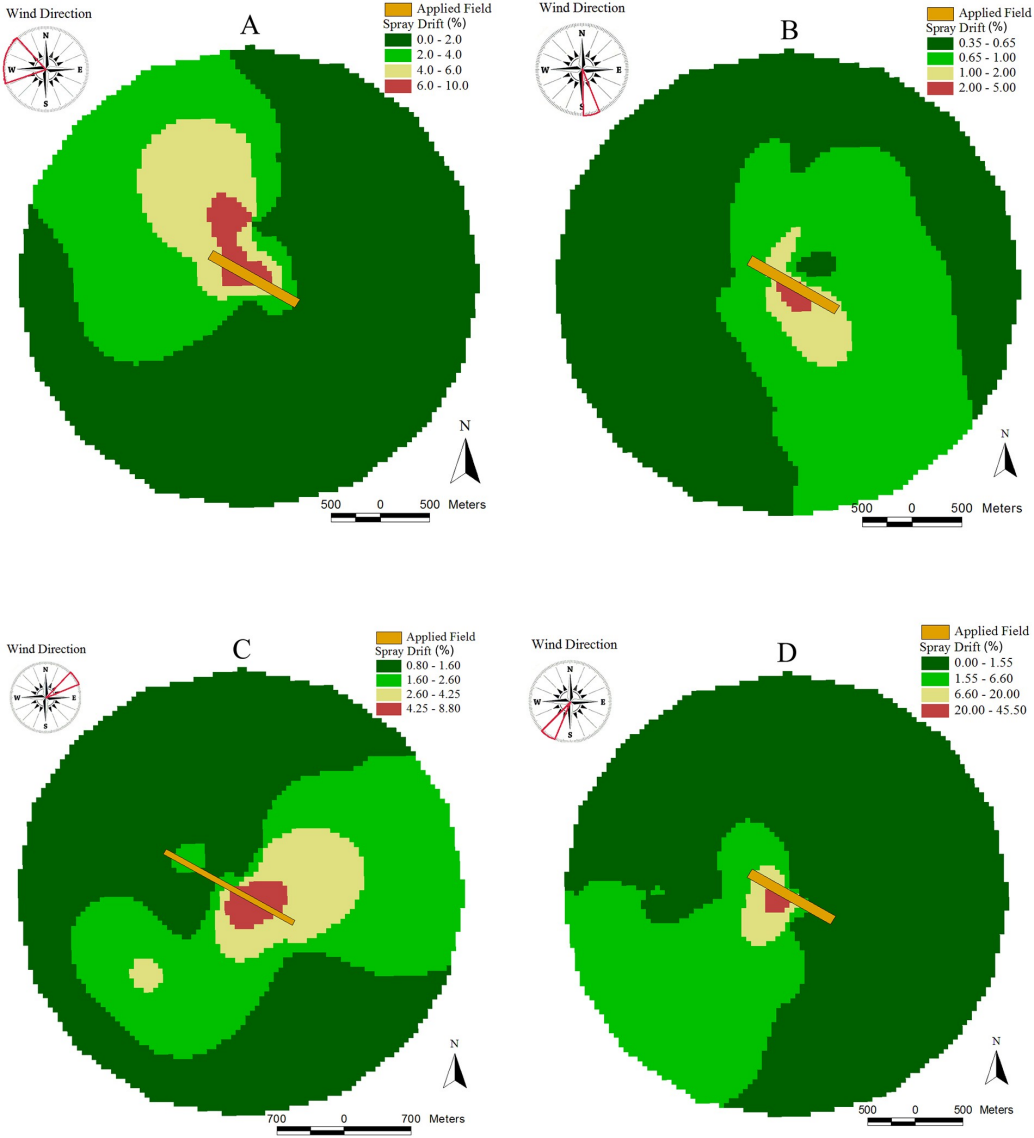
Drift maps (μg m^-2^) from aerial applications related to distances to the center of the field and wind direction: (A) 1st application [mean wind speed—WS = 3.0 km h^-1^]; (B) 3rd application [WS = 7.3 km h^-1^]; (C) 4th application [WS = 14.3 km h^-1^]; and (D) 17th application [WS = 5.5 km h^-1^].

Based on the results of the path analysis, wind speed is the primary climatic variable with a positive cause-and-effect relation with drift, although of low magnitude. The low estimate of the direct effect of wind speed on drift can be explained by the small variation of this variable (Fig 3A). Fig 8 shows the variability of drift spatialization measured at a radius of 2,000 m around the applied field, located in the center of the circle (orange area). The highest drift deposits are concentrated mainly in the predominant direction of the downwind during and after the spray application. When the wind speed was very low (Fig 8A), droplets were suspended in the air for longer by the temperature inversion [35], increasing drift deposits at greater distances from the field border. The droplets that are suspended in the air are blown by the wind after the application [5]. This fact illustrates the high risk of contamination of areas adjacent to the application under unfavorable weather conditions.

## Conclusions

The major pesticide class sprayed with aircraft in the Brazilian Cerrado was insecticide, followed by fungicide. This scenario shows the potential hazard risk of spray drift over the environment.

The concentration of the drift deposits decreased as surrounding distance and application rate were increased.

A mathematical equation of drift prediction was established, where the variables that contributed most to drift deposits were surrounding distance and wind speed. Thus, it is very important to monitor and respect the wind speed limits during the aerial spraying, mainly when there is any risk potential associated with pesticide exposure over the downwind direction.

## Supporting information

S1 Table Data used in experiment. (TXT)
