# Correction: Factors affecting aerial spray drift in the Brazilian Cerrado

**DOI:** 10.1371/journal.pone.0217957

**Published:** 2019-06-04

**Authors:** Fabio Henrique Rojo Baio, Ulisses Rocha Antuniassi, Bruno Rodeguer Castilho, Paulo Eduardo Teodoro, Eder Eujácio da Silva

The following information is missing from the Funding statement: This study was funded in part by the Coordination of Improvement of Higher Education Personnel - Brazil (CAPES) - Finance Code 001; Support from the Federal University of Mato Grosso do Sul.
